# Better diagnostic tools needed to distinguish typhoid from other causes of acute febrile illness

**DOI:** 10.2471/BLT.23.290678

**Published:** 2023-11-01

**Authors:** Sadia Shakoor, Sabine Dittrich

**Affiliations:** a Department of Pathology and Laboratory Medicine, Aga Khan University, Stadium Road, PO Box 3500, Karachi 74800, Pakistan.; b European Campus Rottal Inn, Deggendorf Institut of Technology, Pfarrkirchen, Germany.

More than 8 million people contract typhoid annually, resulting in about 100 000 deaths. Despite associated sociopsychological and economic fallouts,^1^ the typhoid diagnostics field has not accelerated sufficiently to allow reliable detection of this disease.^2^ Co-occurrence of other acute undifferentiated febrile illnesses make clinical examination alone insufficient to differentiate typhoid from other such illnesses, making diagnostic evaluation essential.^3^

The most frequent co-endemic acute undifferentiated febrile illnesses are flaviviral illnesses, scrub typhus, malaria, leptospirosis and acute viral hepatitis.^3^ Evaluations of diagnostics for typhoid and other acute undifferentiated febrile illnesses are complex. Despite the abundance of cases across the world, identifying one setting that allows studies to fulfil sample size requirements for more than one etiology is challenging. The reasons are diverse: pathogen occurrence is often seasonal, and diagnostic accuracy studies are rarely funded to last multiple seasons; and further reference testing can be extensive – to cover various etiologies. Therefore, evaluations are based on highly selected cohorts and do not reflect real-life scenarios from a clinical symptom or co-infection perspective. Various sources of biases are observed in acute undifferentiated febrile illnesses diagnostic test evaluations,^4^ including comparisons with healthy controls only rather than those with similar febrile presentations; variable use of reference tests; and lack of reference samples to determine interlaboratory reproducibility. The result is a lack of representative studies that meet the standards of policy review committees, that could help advise on the appropriate use of diagnostic tests for acute undifferentiated febrile illnesses. The results of a recent study that assessed the performance of commercially available rapid typhoid diagnostic tests highlighted a lack of these tests.^5^ The same study served as the basis of a data dossier submitted to the World Health Organization Strategic Advisory Group of Experts on in vitro diagnostics. The goal was to recommend against the use of these tests and prevent unnecessary use of typhoid rapid diagnostics in high-endemic settings.^6^^,^^7^ The advisory group rejected this proposal, allowing continued use of these tests in endemic settings.^6^ The main argument presented was the unavailability of alternative rapid tests, while acknowledging the suboptimal performance of currently available tests. Whether this decision will fuel further misuse of typhoid rapid diagnostics or provide momentum to typhoid diagnostic test development is debatable; however, the decision highlights a know-do gap in the medical community, where a suboptimal test is preferred to no test. This situation is unfortunate, especially as poor quality medical care can have detrimental outcomes to populations even if they have adequate health-care access.^8^ Meanwhile, the gap in the generalizability of acute undifferentiated febrile illnesses diagnostics to populations of concern, and relevant guidance on algorithmic use of rapid typhoid diagnostic tests, remains unaddressed and needs attention.^9^

Several actionable insights emerge from these gaps that are relevant to future diagnostic test development and evaluations. First, investigators must aim to integrate clinical and laboratory results in diagnostic test evaluations. Clinicians are aware that the diagnosis of acute fever requires a combination of several clinical, physiological and contextual parameters. Systematic assessments of the discriminative power of various tests are important to determine the true added value of rapid tests. Second, researchers should use modelling approaches to identify algorithms worth evaluating in trial settings. Models can inform the incremental value and post-test probabilities of rapid diagnostic tests within clinical algorithms based on pretest accuracies of combinations of clinical signs. To improve testing algorithms for multiple pathogens, the global health community needs renewed data mining efforts to inform modelling approaches. Third, implementers should tailor diagnostic test interventions to the needs and preferences of end-users. Guidelines cannot be translated into interventions unless contextualized to specific practice settings. Qualitative studies should therefore be undertaken in high-burden settings to understand drivers of test use and physician-patient preferences to avoid translation failures. Fourth, funders should incentivize diagnostic research. As new infectious challenges emerge, it is important that funding focus does not shift away from this common cause of illness and death.

The recent World Health Assembly resolution on strengthening diagnostics capacity^10^ is a reminder that diagnostic services are at the heart of surveillance, preparedness and optimal health outcomes. We hope that this call to action will motivate academia and industry to design, evaluate and implement fit-for-purpose tests for acute undifferentiated fever in the global market, to achieve sustainable health impacts.

## References

[1] GBD 2019 Diseases and Injuries Collaborators. Global burden of 369 diseases and injuries in 204 countries and territories, 1990-2019: a systematic analysis for the Global Burden of Disease Study 2019. Lancet. 2020 Oct 17;396(10258):1204–22. 10.1016/S0140-6736(20)30925-9 33069326 PMC7567026

[2] Carey ME, McCann NS, Gibani MM. Typhoid fever control in the 21st century: where are we now? Curr Opin Infect Dis. 2022 Oct 1;35(5):424–30. 10.1097/QCO.0000000000000879 35984009

[3] Wangdi K, Kasturiaratchi K, Nery SV, Lau CL, Gray DJ, Clements ACA. Diversity of infectious aetiologies of acute undifferentiated febrile illnesses in south and Southeast Asia: a systematic review. BMC Infect Dis. 2019 Jul 4;19(1):577. 10.1186/s12879-019-4185-y 31272417 PMC6610835

[4] Kohn MA, Carpenter CR, Newman TB. Understanding the direction of bias in studies of diagnostic test accuracy. Acad Emerg Med. 2013 Nov;20(11):1194–206. 10.1111/acem.12255 24238322

[5] Sapkota J, Hasan R, Onsare R, Arafah S, Kariuki S, Shakoor S, et al. Comparative analysis of commercially available typhoid point-of-care tests: results of a prospective and hybrid retrospective multicenter diagnostic accuracy study in Kenya and Pakistan. J Clin Microbiol. 2022 Dec 21;60(12):e0100022. 10.1128/jcm.01000-22 36448816 PMC9769786

[6] The selection and use of essential in vitro diagnostics: report of the fourth meeting of the WHO Strategic Advisory Group of Experts on In Vitro Diagnostics, 2022, TRS 1053. Geneva: World Health Organization; 2023. Forthcoming.

[7] The selection and use of essential in vitro diagnostics, TRS 1031. Geneva: World Health Organization; 2021. Available from: https://www.who.int/publications/i/item/9789240019102 [cited 2023 Oct 11].

[8] Kruk ME, Gage AD, Arsenault C, Jordan K, Leslie HH, Roder-DeWan S, et al. High-quality health systems in the Sustainable Development Goals era: time for a revolution. Lancet Glob Health. 2018 Nov;6(11):e1196–252. 10.1016/S2214-109X(18)30386-3 30196093 PMC7734391

[9] Pokharel S, White LJ, Aguas R, Celhay O, Pellé KG, Dittrich S. Algorithm in the diagnosis of febrile illness using pathogen-specific rapid diagnostic tests. Clin Infect Dis. 2020 May 23;70(11):2262–9. 10.1093/cid/ciz665 31313805 PMC7245147

[10] Samarasekera U. Diagnostics get global recognition. Lancet. 2023 May 27;401(10390):1758. 10.1016/S0140-6736(23)01044-9 37245513

